# Predicting the risk of endometriosis in Chinese infertile women: development and assessment of a predictive nomogram

**DOI:** 10.3389/fsurg.2025.1541667

**Published:** 2025-11-26

**Authors:** Chenman Wang, Yicheng Li, Wanqing Zhu, Xia Li, Man Wang, Huili Liu

**Affiliations:** 1 Department of Gynecology, People’s Hospital of Zhengzhou University, Zhengzhou, China; 2 Department of Gynecology, Henan Provincial People’s Hospital, Zhengzhou, China; 3 Department of Gynecology, People’s Hospital of Henan University, Zhengzhou, China

**Keywords:** endometriosis, infertility, LASSO, nomogram, prediction model

## Abstract

**Purpose:**

This study aimed to establish a risk prediction model of endometriosis in infertile women and verify the model.

**Methods:**

A retrospective study was made of 140 infertile women hospitalized at Henan Provincial People's Hospital between January 2018 and May 2024. They were divided into the Endometriosis group (EMs) and the No Endometriosis group (No-EMs). The baseline characteristics of the two groups were compared. The least absolute shrinkage and selection operator (LASSO) regression model was utilized to optimize feature selection. Subsequently, logistic regression (LR) analysis was utilized to formulate a predictive model that integrated the selected features. The discrimination and calibration of the predictive model were evaluated using the C-index and calibration plot. Internal validation was conducted using bootstrapping methods.

**Results:**

The LASSO regression model identified five feature selections: menstrual pattern, menstrual cycle length, severity of dysmenorrhea, duration of infertility, and type of infertility. LR analysis revealed that the severity of dysmenorrhea (OR = 10.278, 95% CI = 2.372–73.400, *p* = 0.005) and the type of infertility (OR = 2.604, 95% CI = 1.247–5.563, *p* = 0.012) emerged as independent risk factors for EMs in infertile women. The model displayed good discrimination with a C-index of 0.743 (95% CI = 0.660–0.826)and good calibration. Internal validation through the Bootstrap method confirmed a high C-index value of 0.709.

**Conclusion:**

The development of Nomogram prediction models offers significant clinical predictive utility in evaluating the risk of EMs among infertile women. It equips clinicians with rational treatment strategies and novel perspectives for managing infertile women.

## 1 Introduction

Endometriosis (EMs) is a common gynecological disease that affects women of reproductive age, primarily characterized by the growth and infiltration of endometrial tissue outside the uterine cavity, leading to symptoms such as pain, nodules, masses, and infertility (1–3). Research indicates that the infertility rate among EMs patients is approximately 40.5%, while the incidence of EMs can be as high as 50% among infertile women (4, 5). Although the exact cause of EMs is not completely understood, the widely accepted theory is Sampson's “theory of ectopic implantation.” Hemmert et al. (6, 7) have shown that the occurrence of EMs is associated with various internal and external risk factors, including genetics, endocrine, microorganisms, and environmental factors. These factors may influence the occurrence of EMs by affecting hormone levels in the body. Furthermore, the mechanisms by which EMs lead to infertility are quite complex, seemingly affecting various parts of the female reproductive system (8). Research literature suggests that EMs may cause pelvic adhesions and changes in anatomical structures, thereby impacting ovarian ovulation, oocyte retrieval, and transport capabilities, as well as reducing the receptivity of the endometrium, ultimately leading to infertility and spontaneous pregnancy loss (9, 10).

In clinical practice, laparoscopy is the primary method for diagnosing EMs. However, patients are often reluctant to undergo the procedure due to its invasive nature, surgical risks, and high costs (11). Currently, there is a lack of reliable predictive factors for EMs in clinical settings. Therefore, the goal of establishing a risk prediction model is to assist clinicians in better predicting which infertile women are at high risk for EMs, thereby more effectively determining which patients should undergo laparoscopy to address infertility caused by EMs (12). Some published studies have identified primary infertility, moderate to severe dysmenorrhea, and uterosacral ligament/Douglas nodules as significant independent predictive factors for EMs (12). For obvious adnexal masses and tenderness in the Douglas pouch, experienced clinicians can make a preliminary diagnosis of EMs through imaging and gynecological examinations. However, for early-stage EMs lesions, there are currently no effective diagnostic methods available through imaging and gynecological examinations aside from laparoscopy. Despite significant improvements in imaging technology in recent years, studies have shown that even the most experienced physicians cannot accurately identify or exclude superficial EMs through magnetic resonance imaging (MRI) and ultrasound (13). In our study, we used logistic regression (LR) to determine which clinical factors (including symptoms, menstrual cycle characteristics, and demographic variables) are independent predictive factors for EMs in infertile women.

## 2 Materials and methods

### 2.1 General materials

In this retrospective study, medical records from Henan Provincial People's Hospital between 2018 and 2024 were reviewed. A total of 140 infertile women who underwent their first combined laparoscopic and hysteroscopic surgery were included, and the same experienced gynecologist performed the surgery.

#### 2.1.1 Inclusion criteria

1. Women with a desire for fertility, engaging in normal sexual intercourse, and not using contraception for 1 year or more without achieving pregnancy.
2. No clear preoperative diagnosis of EMs, confirmed through laparoscopic surgery performed at Henan Provincial People's Hospital (from January 2018 to May 2024), with complete medical records available.
3. Surgery performed by the same experienced gynecologist, with postoperative diagnosis confirmed by histopathological examination.

#### 2.1.2 Exclusion criteria

1. Incomplete medical records.
2. Presence of reproductive tract anomalies (uterine septum, uterine malformations) or conditions such as intrauterine adhesions and ovarian function decline that may affect infertility or contribute to miscarriage.
3. Patients with adenomyosis or a history of pelvic inflammatory disease, pelvic infections, or other conditions that may cause pain.
4. Patients with malignant tumors.
5. Male partner with abnormal semen analysis.
6. Previous sterilization surgery, with the current admission requiring tubal reanastomosis.

### 2.2 Methods

A retrospective analysis method was used to collect potential predictive variables that may affect the occurrence of EMs in infertile women. This information was gathered by a single physician through the clinical medical record system and included 16 predictive variables: symptoms [dysmenorrhea (yes/no), severity of dysmenorrhea (none, mild: tolerable without oral analgesics, moderate: requires oral analgesics, severe: oral analgesics do not relieve pain)]; presence of polyps detected during hysteroscopic examination (yes/no); menstrual cycle characteristics [menstrual pattern (regular/irregular), age at menarche, menstrual cycle length and duration of bleeding menstrual]; and demographic characteristics [age, age at marriage, type of infertility (primary/secondary), duration of infertility, number of pregnancies, number of births, history of live births (yes/no), previous abortion (yes/no), and ectopic pregnancy (yes/no)]. All suspected EMs lesions were surgically removed by the same experienced gynecologist, regardless of whether they were typical or atypical lesions under laparoscopy, and the diagnosis was confirmed histologically by the pathology department of our hospital. Based on postoperative diagnoses and pathological results, patients were divided into two groups, the EMs group and the No-EMs group, and the influencing factors of both groups were compared.

### 2.3 Statistical methods

This study utilized SPSS 22.0 statistical software for data analysis. In the univariate analysis, count data were expressed as *n* (%), while other data were presented as mean ± standard deviation (minimum and maximum), and baseline charts were drawn. Statistical analyses were conducted using R software (version 4.4.1; https://www.r-project.org/). The least absolute shrinkage and selection operator (LASSO) regression analysis was used to identify potential risk factors, and the selected variables were incorporated into the model using the assignment method. The logistic regression (LR) model was used to analyze the influencing factors, thereby identifying independent risk factors for infertility patients with EMs. We constructed a nomogram model using the nomogram method from the “rms” package. We plotted the receiver operating characteristic (ROC) curve to evaluate the model's discriminatory ability and plotted the calibration curve to assess and calibrate the model, with internal validation performed using the Bootstrap method. All processes were completed in R software (version 4.4.1), and statistical significance was defined as *p*-value <0.05.

## 3 Results

### 3.1 Patients' characteristics

From January 2018 to May 2024, a total of 140 eligible study subjects were collected from medical records. Among them, there were 71 patients without EMs and 69 patients with EMs. The average age of the No-EMs group was (30.3 ± 5.13) years, while the average age of the EMs group was (29.1 ± 14.09) years. The age range of the total participants was (18–41) years. The analysis revealed no statistically significant differences between the two groups in terms of age, age at marriage, age at menarche, menstrual pattern, menstrual cycle length, duration of menstruation, polyps, previous abortion, and ectopic pregnancy (*p* > 0.05). However, there were statistically significant differences in the number of pregnancies, number of births, history of live births, dysmenorrhoea, severity of dysmenorrhoea, duration of infertility, and type of infertility (*p* < 0.05) (see Table 1).

**Table 1:** Compare the baseline characteristics between the EMs and the No-EMs group.

Variables	No endometriosis (*N* = 71)	Endometriosis (*N* = 69)	Total (*N* = 140)	*p*-Value
Age (years)				
Mean (SD)	30.3 (5.13)	29.1 (4.09)	29.7 (4.67)	0.421
Median [Min, Max]	30.0 [21.0, 41.0]	29.0 [18.0, 41.0]	29.0 [18.0, 41.0]	
Age at marriage (years)				
Mean (SD)	24.1 (4.10)	25.1 (3.30)	24.6 (3.75)	0.090
Median [Min, Max]	24.0 [16.0, 38.0]	25.0 [16.0, 33.0]	25.0 [16.0, 38.0]	
Age at menarche (years)				
Mean (SD)	13.3 (1.32)	13.2 (1.38)	13.2 (1.34)	1
Median [Min, Max] Number of pregnancles	13.0 [11.0, 16.0]	13.0 [10.0, 18.0]	13.0 [10.0, 18.0]	
Mean (SD)	1.04 (1.18)	0.565 (0.899)	0.807 (1.07)	0.021
Median [Min, Max]	1.00 [0, 4.00]	0 [0, 3.00]	0 [0, 4.00]	
Number of births				
Mean (SD)	0.437 (0.603)	0.217 (0.481)	0.329 (0.555)	0.046
Median [Min, Max]	0 [0, 2.00]	0 [0, 2.00]	0 [0, 2.00]	
History of live births				
No	44 (62.0%)	56 (81.2%)	100 (71.4%)	0.043
Yes	27 (38.0%)	13 (18.8%)	40 (28.6%)	
Menstrual pattern				
Regular	57 (80.3%)	62 (89.9%)	119 (85.0%)	0.284
Irregular	14 (19.7%)	7 (10.1%)	21 (15.0%)	
Menstrual cycle length (days)				
Mean (SD)	31.5 (9.37)	29.5 (3.89)	30.5 (7.25)	0.557
Median [Min, Max]	30.0 [22.0, 90.0]	29.0 [22.5, 45.0]	30.0 [22.0, 90.0]	
Duration of bleeding menstrual (days)				
Mean (SD)	5.72 (1.81)	5.60 (1.26)	5.66 (1.56)	0.940
Median [Min, Max] Polyps	6.00 [2.50, 15.0]	5.50 [3.00, 8.00]	6.00 [2.50, 15.0]	
No	62 (87.3%)	59 (85.5%)	121 (86.4%)	0.952
Yes	9 (12.7%)	10 (14.5%)	19 (13.6%)	
Dysmenorrhea				
No	46 (64.8%)	29 (42.0%)	75 (53.6%)	0.026
Yes	25 (35.2%)	40 (58.0%)	65 (46.4%)	
Severity of dysmenorrhea				
None	46 (64.8%)	28 (40.6%)	74 (52.9%)	0.016
Mild	23 (32.4%)	29 (42.0%)	52 (37.1%)	
Moderate	2 (2.8%)	12 (17.4%)	14 (10.0%)	
Duration of infertility (Y)				
Mean (SD)	2.99 (2.76)	2.20 (2.28)	2.60 (2.56)	0.016
Median [Min, Max]	2.00 [1.00, 17.0]	1.00 [1.00, 12.0]	2.00 [1.00, 17.0]	
Type of infertility				
Primary	29 (40.8%)	45 (65.2%)	74 (52.9%)	0.015
Secondary	42 (59.2%)	24 (34.8%	66 (47.1%)	
Previous abortion				
No	46 (64.8%)	54 (78.3%)	100 (71.4%)	0.211
Yes	25 (35.2%)	15 (21.7%)	40 (28.6%)	
Ectopic pregnancy				
No	66 (93.0%)	66 (95.7%)	132 (94.3%)	0.790
Yes	5 (7.0%)	3 (4.3%)	8 (5.7%)	

SD, standard deviation; Min, minimum; Max, maximum. After counting all the data, there were no patients with severe dysmenorrhea found, so they were not included in the table.

### 3.2 Selection of factors associated with infertility-related-EMs using the LASSO regression model

**Figure 1:**
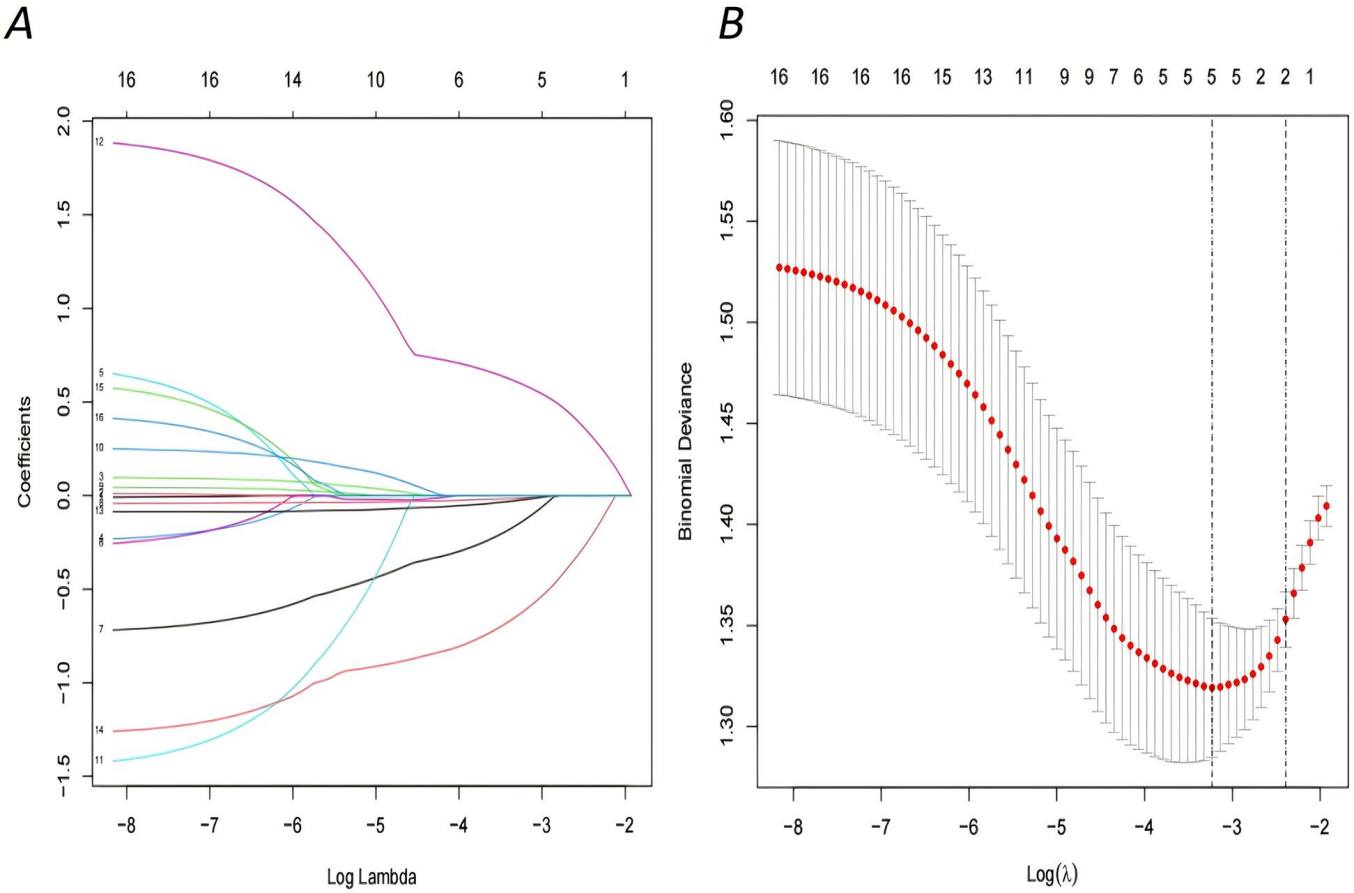
Demographic and clinical feature selection using the LASSO regression model. **(A)** LASSO coefficient profiles of the 16 features.A coefficient profile plot was produced against the log (lambda) sequence, optimal lambda resulted in five features with nonzero coefficients. **(B)** The selection of the optimal parameter within the LASSO model was executed through a five-fold cross-validation approach based on the minimum criterion. Vertical dotted lines were drawn at optimal values using the minimum criteria and its 1 SE (1—SE criteria).

Based on the collected 16 variables (age, age at marriage, age at menarche, number of pregnancies, number of births, history of live births, previous abortion, ectopic pregnancy, menstrual pattern, menstrual cycle length, duration of bleeding menstrual, polyps, dysmenorrhoea, severity of dysmenorrhoea, duration of infertility, type of infertility), the LASSO regression model was implemented to filter the feature variables. When the penalty coefficient was set to *λ*.1se (0.0395), a total of 5 optimal variables were selected (menstrual pattern, menstrual cycle length, severity of dysmenorrhoea, duration of infertility, type of infertility) (see Figure 1).

### 3.3 LR analysis of factors influencing infertility-related EMs

The five factors selected by the LASSO regression model include: menstrual regularity (coded as “irregular” = 0, “regular” = 1), menstrual cycle length (coded as “>30 days” = 0, “<=30 days” = 1), the severity of dysmenorrhea (coded as “none” = 0, “mild” = 1, “moderate” = 2), duration of infertility (coded as “>2 years” = 0, “<=2 years” = 1), and type of infertility (coded as “secondary” = 0, “primary” = 1). These factors were used as independent variables, with the presence of EMs in infertility patients as the dependent variable (coded as “No-EMs” = 0, “EMs” = 1) for a LR analysis. The results indicated that the severity of dysmenorrhea and type of infertility are independent risk factors for the presence of EMs in infertility patients (*p* < 0.05) (see Table 2).

**Table 2:** Logistic regression analysis of factors influencing infertility-related EMs.

Variables	*β*	SE	OR	95% CI	*p*-Value
Intercept	−2.488	0.719	0.083	0.0186–0.317	<0.001
menstrual pattern	0.594	0.625	1.812	0.543–6.517	0.342
menstrual cycle length	0.724	0.575	2.063	0.676–6.627	0.208
mild dysmenorrhea*	0.583	0.392	1.791	0.831–3.890	0.138
moderate dysmenorrhea*	2.330	0.838	10.278	2.372–73.400	0.005
duration of infertility	0.597	0.424	1.817	0.798–4.249	0.159
type of infertility	0.957	0.380	2.604	1.247–5.563	0.012

SE, standard erro; OR, Odds ratio; CI, confidence interval. *β* is the regression coefficient.

* Represents taking none dysmenorrhea as a reference.

### 3.4 Developed infertility-related EMs nomogram

**Figure 2:**
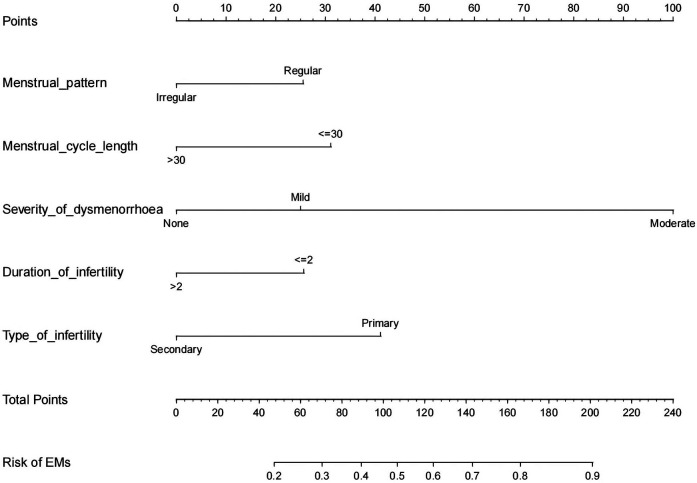
Developed infertility-related EMs nomogram. Add up the scores obtained by drawing vertical lines upward from the endpoints of each predictor variable, and thus the total score can be finally obtained. Then draw a vertical line downward from the total score corresponding to the predicted probability at the bottom to get the risk of EMs occurrence.

Based on LR analysis, we incorporated the two independent risk factors mentioned above, along with menstrual pattern, menstrual cycle length, and duration of infertility, to develop a nomogram prediction model for assessing the risk of EMs in infertility women. The figure clearly illustrates that as the severity of dysmenorrhea increases, the risk of developing EMs also rises. Furthermore, patients with primary infertility are more likely to have EMs compared to those with secondary infertility (see Figure 2).

### 3.5 Evaluation and validation of the nomogram prediction model

**Figure 3:**
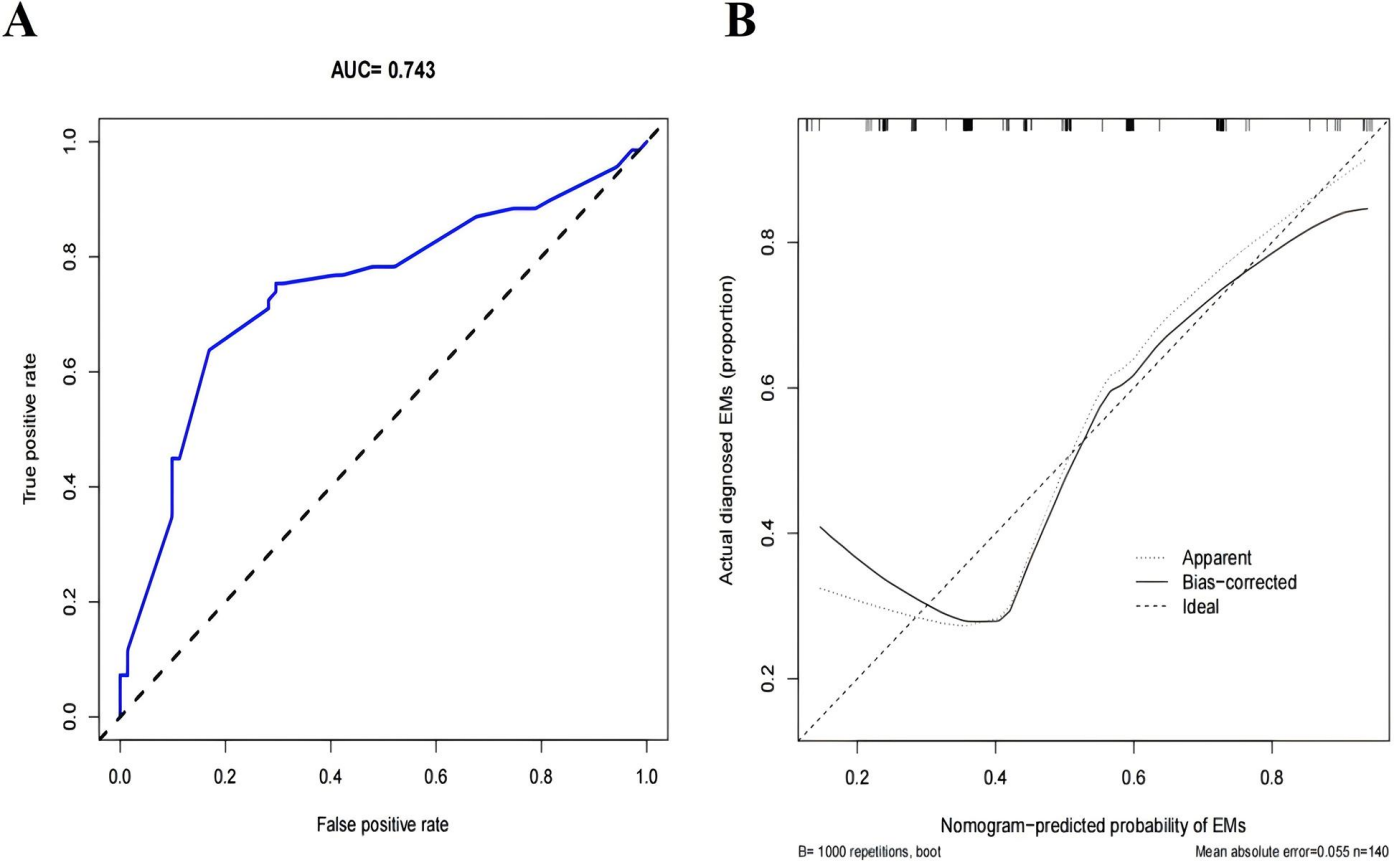
Evaluation and validation of the nomogram prediction model. **(A)** The ROC curve of the nomogram prediction model. **(B)** Calibration curves of the Infertility-Related EMs prediction in the cohort, The *x*-axis represents the predicted EMs risk, the *y*-axis represents the actual diagnosed EMs. The diagonal dotted line symbolizes the flawless predictions generated by an ideal model. The solid line depicts the performance of the nomogram, and the nearer it is to the diagonal dotted line, the more precise the prediction it implies.

We evaluated the performance of the Nomogram prediction model using the ROC curve, which showed an AUC of 0.743 (95% CI = 0.660–0.826), indicating that the model has moderate accuracy (Figure 3A). In addition, we plotted the calibration curve to assess and calibrate the model (Figure 3B). The results of internal validation using the Bootstrap method (1,000 bootstrap resamples) showed a C-index of 0.709, further confirming the model's robust predictive capability.

## 4 Discussion

Infertility often has profound long-term or short-term effects on patients and their families, exposing them to familial and social pressures. According to estimates from the World Health Organization (WHO), 8%–12% of couples of reproductive age worldwide are affected by infertility (14). Recent research by Van Gestel et al. (15) indicates that 44% of women with unexplained infertility are diagnosed with EMs. The results of this study show that among 140 patients who underwent laparoscopic exploration for infertility, 69 cases (49%) were diagnosed with EMs, a proportion consistent with findings from related studies (4, 5, 16). The prevalence of EMs in patients with primary infertility is 65.2% (45/69, *p* = 0.0154), which is significantly higher than 34.8% (24/69) in those with secondary infertility.

In this study, the average values for age, age at marriage, and age at menarche did not demonstrate statistical significance (Table 1). The research conducted by Shafrir et al. (7) indicated that an earlier age at menarche is associated with a higher risk of EMs; however, the findings from Ashrafi et al. (17) did not establish a significant association between age at menarche and EMs. Apparently, there is some controversy regarding the age at menarche for the comorbidity of EMs in infertile women.

The number of pregnancies and births and history of live births were statistically different in the univariate analysis (*p* < 0.05) (Table 1); 27 patients in the No-EMs group had a history of live births, and only 13 patients in the EMs group possessed a history of live births, which clearly showed a negative correlation between the history of live births and EMs. Our data also examined the mean number of pregnancies in patients with No-EMs (1.04 ± 1.08) as compared to the mean number of pregnancies in patients with EMs (0.57 ± 0.90), which suggests that the number of pregnancies is negatively correlated with EMs, which is in line with the study by Ashrafi (17). It has been demonstrated that pregnancy causes the disease to subside in many women, while more births reduce the risk of EMs, and the risk of EMs is increased in childless individuals (18). The mechanism of EMs is still unclear, and several studies have shown that the development of EMs may be associated with abnormal expression of estrogen and progesterone receptors (19, 20). It has also been shown to be because EMs is an estrogen-dependent, progesterone-resistant disease (21). During pregnancy, progesterone levels in women are significantly elevated. This hormone has an antagonistic effect on estrogen, inhibiting endometrial hyperproliferation and thereby greatly reducing the occurrence of EMs. Consequently, this suggests that women who experience more pregnancies are less likely to suffer from EMs.

In this study, there was no statistically significant difference between the two groups in terms of menstrual pattern, menstrual cycle length, and duration of menstruation. However, Moini et al. (22) concluded that menstrual cycle length was negatively correlated with the risk of EMs, whereas irregularity of menstrual, duration, and amount of menstrual bleeding were only associated with severe EMs. Since this study did not conduct research and exploration on the severity grading of EMs, the correlation between infertile women with EMs and menstrual characteristics was not obtained. Also, considering that each woman has a different understanding of bleeding, it was not included in the study. Most studies have shown that shorter menstrual cycles with longer bleeding time and bleeding volume are associated with increased EMs (22, 23), and the findings of the above scholars are in line with our most commonly cited theory of menstrual blood reflux in the formation of EMs.

In this study, no significant correlation was found between the presence of polyps and EMs (*p* > 0.05). Zhang et al. (24) discovered that the prevalence rate of endometrial polyps (EPs) was remarkably elevated in infertile women who had EMs in contrast to those without EMs (*p* < 0.001). This discrepancy may be attributed to the larger sample size in Zhang's study, which included all infertile women undergoing hysteroscopy and laparoscopy at the hospital, whereas our study had a relatively smaller sample size as all surgeries were performed by the same gynecologist, thus failing to establish a correlation between EMs and polyps. However, existing studies indicate that there is some correlation between EMs and polyps in terms of pathogenesis, risk factors, and treatment. Although the exact pathogenesis remains unclear, histological examinations show that both conditions exhibit excessive proliferation of endometrial tissue. Regarding risk factors, body mass index (BMI) and obstetric history are common risk factors for both. In terms of treatment, oral contraceptives or progestin medications can delay the progression of the disease (25, 26). Therefore, although this study did not clearly identify a correlation between the two, the relationship between EMs and polyps still warrants further exploration.

Our research has concluded that the severity of dysmenorrhea is an independent risk factor for infertile women with EMs. Our study is similar to that conducted by Ashrafi et al. (17). However; there are certain differences compared to the research results of Meuleman et al. Their research findings showed that approximately half (46%) of the patients with pain did not have EMs (16). This might be because their research institution is a tertiary referral center for EMs, and during the referral process, it is possible that patients' memories regarding the frequency and intensity of pain symptoms were exaggerated.

Nomograms are widely used as important predictive tools in oncology and medicine. Such diagrams can present information in a clearer, more intuitive manner, which is easier to understand and more convenient for clinical application. The predictive nomogram we developed regarding whether infertile women have EMs or not has demonstrated good discriminative ability and calibration performance in the internal validation of the cohort. In particular, the high C-index in the interval validation indicates that it is helpful for the personalized prediction of whether infertile women have EMs or not. Based on the prediction results, the next step of diagnosis and treatment plans can be formulated, such as whether to conduct laparoscopic exploration or adopt other treatment options like assisted reproduction.

### 4.1 Advantages and limitations

Compared with domestic and foreign studies, this study only retrieved infertility surgeries performed by the same gynecologist, removed the existing EMs lesions in the pelvic cavity, and confirmed the diagnosis histologically, and there were detailed medical record data. Thus, the difference in subjective judgment of EMs lesions was excluded. This study only investigated some infertile women in mainland China. Compared with foreign studies, ethnic differences were excluded. Our current study has limitations. First, as a retrospective study, there is a sample selection bias, resulting in the results not being representative of the entire target population. Second, the risk factor analysis did not include all potential factors related to EMs, and there is a certain degree of subjective difference in data interrogation. Third, although the robustness of our model has been extensively examined through internal validation, external validation cannot be carried out, and the universality of infertile populations in other regions is still being determined. In future studies, it is advisable to conduct a multicenter analysis, which can not only avoid overfitting the model but also enable external validation, making the model more reasonable. We look forward to future multicenter and prospective large-scale studies on infertile women with EMs.

## 5 Conclusion

This study has developed a new type of nomogram for whether infertile women have EMs, which has relatively high accuracy and can help clinicians fully understand the risk of infertile women with EMs before starting treatment. Our study concludes that the severity of dysmenorrhea and the type of infertility are independent risk factors for infertile women with EMs. Through the estimation of individual risks, clinicians and patients can take more necessary measures in medical interventions. Due to the high incidence of EMs, when women consult about the causes of infertility and the risk factors of EMs, our nomogram will be helpful for the early screening, detection, and prevention of EMs.

## Data Availability

The raw data supporting the conclusions of this article will be made available by the authors, without undue reservation.
